# Separate Effects of Foliar Applied Selenate and Zinc Oxide on the Accumulation of Macrominerals, Macronutrients and Bioactive Compounds in Two Pea (*Pisum sativum* L.) Seed Varieties

**DOI:** 10.3390/plants11152009

**Published:** 2022-08-01

**Authors:** Maksymilian Malka, Gijs Du Laing, Torsten Bohn

**Affiliations:** 1 Laboratory of Analytical Chemistry and Applied Ecochemistry, Faculty of Bioscience Engineering, Ghent University, Coupure Links 653, 9000 Gent, Belgium; maksymilian.malka@ugent.be (M.M.); gijs.dulaing@ugent.be (G.D.L.); 2 Nutrition and Health Research Group, Department of Precision Health, Luxembourg Institute of Health, 1 A-B, Rue Thomas Edison, 1445 Strassen, Luxembourg

**Keywords:** oxidative stress, macrominerals, macronutrients, selenate, zinc oxide, food security, bioactive compounds, nutrition, foliar application

## Abstract

Selenium (Se) and zinc (Zn) are important cofactors for antioxidant enzymes. Foliar Se/Zn application is a highly efficient strategy of plant biofortification. However, its effects on the accumulation of macrominerals, macronutrients and bioactive compounds in the pea plant (*Pisum sativum* L.) have been poorly investigated. A two-year pot experiment was performed to study responses of two pea varieties (Ambassador, Premium) to foliar-applied sodium selenate (0/50/100 g Se/ha) and zinc oxide (0/375/750 g Zn/ha) at the flowering stage. Concentrations of Ca, Mg, K, Na, soluble solids (SSC), protein, chlorophyll a and b, total chlorophyll, total carotenoids and total condensed tannins (TCT) were determined in seeds. Mg concentration in Ambassador and chlorophyll a concentration in Premium were positively affected, in part, by selenate and zinc oxide, respectively. Selenate and zinc oxide increased, in part, protein concentration in Premium. Highest protein concentration was found in Premium treated with 375 g Zn/ha (27.6% DW) vs. the control (26.6% DW). Significant (all *p* < 0.001) positive correlations were found, among others, between concentrations of Zn and Mg (r^2^ = 0.735) and between Zn and protein (r^2^ = 0.437) for Ambassador, and between Mg and protein (r^2^ = 0.682), between Zn and Mg (r^2^ = 0.807), as well as between Zn and protein (r^2^ = 0.884) for Premium. TCT significantly (all *p* < 0.05) and positively correlated with SSC (r^2^ = 0.131), chlorophyll b (r^2^ = 0.128) and total chlorophyll (r^2^ = 0.109) for Ambassador. This study provides new nutritional data on Se/Zn biofortified peas, important for improving agronomic biofortification of pea plants.

## 1. Introduction

Bioactive compounds, also known as plant bioactives or bioactive secondary plant metabolites, are (for humans) non-essential constituents found in small amounts in plants and certain foods (such as fruits and vegetables) that have been shown to have positive effects on human health. Bioactive compounds may be involved in various functions in the plant including growth, development and reproduction, and resistance against biotic and abiotic stress conditions, including herbivores, bacteria and fungi, as reviewed by Loi et al. [1]. Chlorophylls, carotenoids and tannins are examples of plant-derived natural bioactive compounds.

Chlorophyll derivatives have shown, at least in vitro, relevant biological activities consistent with the prevention of cancer, including antioxidant and antimutagenic activity, mutagen trapping, modulation of xenobiotic metabolism and induction of apoptotic events [2,3]. Carotenoids are thought to decrease the risk of several diseases, such as age-related eye disorders, cardiovascular diseases, as well as some types of cancer [4,5]. Tannins are polyphenols that have potential antiviral, antibacterial, enzyme-inhibiting, antioxidant, radical-scavenging and antimutagenic effects, and have thus been proposed to have anti-diabetic and cardio-protective effects [6,7,8].

Pulse crops are essential for sustainable agriculture and environment, biodiversity, global health and food security [9]. Pea (*Pisum sativum* L.) is an important legume and staple crop grown globally and employed for animal and human diets. Peas are very nutritious, providing proteins [10], carbohydrates, fiber, starch, vitamins, minerals and phytochemicals, among others [11,12]. Consumption of peas and pea constituents has been associated with metabolic, cardiovascular and gastrointestinal health benefits in humans [11,13]. It is worth stressing that there is an important gap regarding studies on potential health benefits of individual pea compounds, including proteins [11] and phytochemicals, as well as their mineral concentrations.

Selenium (Se) and zinc (Zn) are trace elements nutritionally essential for humans, and suboptimal Se and Zn statuses are widespread (including Europe), reflecting to a large extent inadequate soil levels [14]. Low Se and Zn statuses have been linked to various health problems including cancer, neurodegenerative diseases, cardio-metabolomic complications (such as diabetes or metabolic syndrome) [15,16] and infectious diseases, including also the recent COVID-19 pandemic [17,18].

Agronomic biofortification has been investigated as a promising strategy to improve the nutritional value (mainly micronutrients) of staple foods. Previous studies have shown that foliar application is a highly effective method for Se and Zn biofortification [19,20]. However, little is known on the influence of such treatments on the accumulation of bioactive compounds in legume crops including pea, which may contribute to health beneficial effects also in humans. Since Se and Zn are involved in many metabolic processes, enhanced Se and Zn availability by the plant may have consequences for bioactive plant compounds.

Therefore, the current study aimed to examine the effect of different doses of foliar-applied Se (sodium selenate) and Zn (zinc oxide) at the flowering stage on two pea varieties (Ambassador and Premium). For this purpose, we studied the accumulation of selected macrominerals, macronutrients and selective bioactive plant metabolites in seeds, and then investigated their relation to antioxidant activity. Finally, their respective correlations were also assessed.

## 2. Materials and Methods

### 2.1. Chemicals

Zinkuran SC was purchased from Arysta LifeScience Slovakia s.r.o. (Nové Zámky, Slovakia). Sodium selenate was obtained from Alfa Aesar (Karlsruhe, Germany). Nitric acid (HNO_3_, for mineral analysis) and hydrogen peroxide 30% (Suprapur) were purchased from LGC Standards (Molsheim, France) and Merck/VWR (Leuven, Belgium), respectively. Methanol was purchased from Biosolve (Valkenswaard, the Netherlands). Acetone was obtained from Merck (Darmstadt, Germany). Vanillin and (+)-catechin were purchased from Sigma-Aldrich (St. Louis, MO, USA). Hydrochloric acid (HCl) was obtained from VWR (Leuven, Belgium).

### 2.2. Design of Experiment and Sample Preparation

A two-year outdoor pot experiment was conducted in the Botanical Garden of the Slovak University of Agriculture in Nitra (48.305 N, 18.096 E), located in Nitra, Slovakia, from March to June 2014/15. For this purpose, we had four replicates for each treatment with 2 pea varieties, which were studied during 2 years and with a total of 5 treatments (i.e., with a total of 80 pots), i.e., 3 main factors were investigated. During the experiment, the average monthly temperature and precipitation in the 2014/15 growing season were recorded, which were: March (9.3 °C and 15.4 mm), April (12.4 °C and 48.9 mm), May (15.2 °C and 57.6 mm) and June (19.3 °C and 52.5 mm), and in the 2015 growing season were as follows: March (6.3 °C and 35.4 mm), April (10.4 °C and 25.0 mm), May (15.1 °C and 69.5 mm) and June (19.9 °C and 10.2 mm). The experimental soil was gleyic fluvisol. The soil from the 2014 season had a pH of 6.47 and contained 19.5 mg kg^−1^ of N, 86.3 mg kg^−1^ of P, 498 mg kg^−1^ of K, 6610 mg kg^−1^ of Ca, 816 mg kg^−1^ of Mg, 26.25 mg kg^−1^ of S, 2.47 mg kg^−1^ of Zn, 0.08 mg kg^−1^ of Se and 3.46% of humus. The soil from the 2015 season had a pH 7.16 and contained 19.1 mg kg^−1^ of N, 245 mg kg^−1^ of P, 150 mg kg^−1^ of K, 6340 mg kg^−1^ of Ca, 644 mg kg^−1^ of Mg, 7.5 mg kg^−1^ of S, 2.39 mg kg^−1^ of Zn, 0.08 mg kg^−1^ of Se and 3.25% of humus. Seeds of two high-yielding and dark-seeded *P. sativum* varieties, i.e., Ambassador (late variety) and Premium (early variety), were provided by a local farmer. Soil-filled plastic square pots (10 L) were placed in a wire mesh housing to protect plants against bird attacks. Pea sowing (thirty seeds/pot sown in two rows at 5 cm depth) was performed in mid-March. Se as sodium selenate and Zn as Zinkuran SC (30% ZnO + 6% chelate) were separately examined by the application of the five following treatments: un-amended control (control), 50 g Se/ha (Se1), 100 g Se/ha (Se2), 375 g Zn/ha (Zn1) and 750 g Zn/ha (Zn2). Se and Zn treatments were applied via foliar spraying at the flowering stage of plants during non-rainy periods. A manual sprayer was employed for the application of fertilizers. No additional fertilization was used. During the experiment, regular irrigation and phytosanitary control were used. There was no toxic effects of foliar Se and Zn applications on plants or incidences of pests and diseases. Freshly harvested seeds at physiological maturity were used to measure soluble solids concentration. The rest of the seeds were then immediately lyophilized, homogenized by grinding, and Ca, Mg, K, Na, protein, chlorophyll a, chlorophyll b, total chlorophyll, total carotenoid and total condensed tannin concentrations were examined.

### 2.3. Concentration of Ca, Mg, K and Na

An aliquot (0.2 g) of each sample was mixed with 3.5 mL HNO_3_ (65%) and 3.5 mL H_2_O_2_ (30%). Thereafter, microwave digestion for complete combustion of organic matrix was carried out using a MARS 6 system (CEM, Orsay, France, 1200 W, 10 min at 55 °C; 10 min at 75 °C; and 45 min at 120 °C). Ca, Mg, K and Na concentrations (mg/kg dry weight (DW)) were subsequently determined in the diluted digests via ICP-OES (Varian Vista MPX, Palo Alto, CA, USA). External calibration was used. Accuracy and precision were monitored by periodic evaluation of a calibration blank, re-analyzing standards during sample runs, and analysis of certified reference materials (including rice flour NIST1568a, sea lettuce BCR279 and spinach leaves SRM 1570a), spiked samples and analytical duplicates.

### 2.4. Soluble Solids and Protein Concentration

Soluble solids concentration (SSC) was measured using a digital handheld refractometer (DR201-95, A. KRÜSS Optronic, Hamburg, Germany). Each sample was examined in two replicates, and the mean was used as the SSC value (% fresh weight (FW)).

The protein concentration was determined via the Dumas method with the TruSpec CHNS analyzer (LECO, Saint Joseph, MI, USA). Analyses were carried out as duplicates. The results were then multiplied by the nitrogen conversion factor for legume protein −5.4 [21] to obtain the protein concentration in the examined samples according to the formula:

nitrogen concentration [% DW] × 5.4 = total protein concentration [% DW](1)

### 2.5. Extraction and Determination of Chlorophylls and Carotenoids

According to the procedure of Kaulmann et al. [22], 100 mg of lyophilized pea material was weighted into a 15 mL falcon tube. Samples were then mixed with 2 mL of acetone, vortexed (1 min), sonicated (5 min), put on ice for 5 min and centrifuged for 2 min at 2500 g and 4 °C. The supernatant was transferred to a 50 mL falcon tube. The residue was re-extracted four times with 2 mL of acetone as described above without ice application. Supernatants were then combined and mixed briefly, and the volume of the acetone phase was noted down. An aliquot was then filtered through a 0.45 um filter. Unused samples were stored under Argon at −80 °C.

The following formulas were used to calculate the concentration of chlorophyll a and b and the total carotenoid concentration [23]:

chla (µg/mL) = (11.24 A663 − 2.04 A645)(2)

chlb (µg/mL) = (20.13 A645 − 4.19 A663)(3)

c(µg/mL) = (1000 A470 − 1.90 chla − 63.14 chlb)/214(4)

where: A = absorbance, chla = concentration of chlorophyll a (mg/100 g DW), chlb = concentration of chlorophyll b (mg/100 g DW), c = total carotenoid concentration (mg/100 g DW).

### 2.6. Extraction and Determination of Total Condensed Tannins

The protocol produced by Bouayed et al. [24] was used for the extraction of tannins from dry material. For this purpose, 0.5–1 g of lyophilized pea material was weighed in a 15 mL screw-cap falcon tube and mixed with 7.5 mL of 80% (*v*/*v*) methanol. The mixture was then sonicated for 5 min and centrifuged at 4000 g for 5 min (Heraeus Multifuge X3, Thermo Scientific, Leuven, Belgium). The supernatant was transferred into a new 15 mL screw-cap falcon. The residue from the first falcon tube was re-extracted with 3 mL of 80% methanol, mixed, sonicated (5 min) and centrifuged at 4000 g for 5 min. Supernatants were combined and this step was repeated once more. To evaporate the methanol, the combined extract was treated with a stream of nitrogen in a Turbovap blower (Caliper Life Sciences, Teralfene, Belgium) until approx. two mL remained (if necessary, an appropriate amount of distilled water was added to reach an even number). Methanol (100%) was then added to reach a final volume of 4 mL. The extracts were stored under argon at −80 °C until further examination.

A modified method of Sun et al. [25] was used to determine the concentration of total condensed tannins. Toward this end, 100 µL of appropriately diluted extract or standard was mixed with 1.5 mL of 4% vanillin solution in methanol and 0.75 mL of concentrated (32%) HCl. The mixture was incubated in the dark at 35 °C for 15 min. After this, the absorbance was measured at 500 nm vs. the blank. An external calibration curve (catechin, *n* = 6 concentrations between 0 and 150 mg/L) was prepared to determine the total condensed tannin concentration expressed as catechin equivalents (CE) per 100 g DW.

### 2.7. Statistical Analysis

Normal distribution of data and equality of variance were verified by normality plots and box plots, respectively. Whenever required, data were log-transformed in order to achieve normal distribution. Multivariate models were then employed, with macromineral/macronutrient/bioactive plant compound concentrations as the observed (dependent) variables, and genetic variant, year and biofortificant type (Se or Zn) as independent, fixed factors. Fortification levels were nested within biofortificant. Following significant Fisher F tests, all group-wise comparisons were carried out (Bonferroni post hoc tests). A *p*-value of < 0.05 was considered statistically significant (2-sided). SPSS version 19.0 (IBM, Chicago, IL, USA) was used for all analyses including correlation analyses.

## 3. Results

### 3.1. Overall Effects

Combined analysis of variance for pea seed macrominerals, macronutrients and bioactive compounds showed that treatment significantly affected only soluble solid and protein concentrations. Growing year had a significant effect on Ca, Mg, K, Na, protein, chlorophyll a and total chlorophyll concentrations. Variety showed a significant effect on all variables except for soluble solid, chlorophyll b and total condensed tannin concentrations, and a trend for Na concentration. Interactions were significant in some cases including trends (Table 1).

### 3.2. Ca, Mg, K and Na Concentrations in Seeds

In 2014, treatment did not significantly affect Ca concentration vs. controls in both varieties. In 2015, treatment failed to show a significant influence (*p* = 0.050) on Ca concentration vs. the control in Premium, while the Zn1 treatment significantly increased Ca concentration vs. Se1 and Se2 treatments in Ambassador. For both years, Premium showed significantly higher Ca concentration than Ambassador for all treatments. Growing year had a significant effect on Ca concentration in Premium compared with Ambassador (Table 2).

In 2014, treatment did not significantly influence Mg concentration vs. controls in both varieties. Ambassador showed significantly higher Mg concentration than Premium only for the control. In 2015, the Se1 treatment significantly increased Mg concentration vs. the control in Ambassador, while both Se and Zn treatments significantly decreased Mg concentration vs. the control in Premium. Premium showed significantly higher Mg concentration than Ambassador only for the control. In contrast, Ambassador showed significantly higher Mg concentration than Premium for Se1, Zn1 and Zn2. Growing year had a significant effect on Mg concentration in both varieties (Table 2).

In 2014, treatment did not significantly affect K concentration vs. controls in both varieties. Ambassador showed significantly higher K concentration than Premium for all treatments. In 2015, treatment did not significantly influence K concentration vs. the control in Ambassador, while the Zn2 treatment significantly decreased K concentration vs. the control in Premium. Ambassador showed significantly higher K concentration than Premium for all treatments except for Zn1. Growing year had no significant effect on K concentration in both varieties (Table 2).

For both years, treatment did not significantly affect Na concentration vs. controls in Ambassador and Premium. Premium showed significantly higher Na concentration than Ambassador only for the control and Zn2 in the 2015 growing season. Growing year had a significant effect on Na concentration in Ambassador compared with Premium (Table 2).

### 3.3. Soluble Solids Concentration (SSC) in Seeds

**Figure 1:**
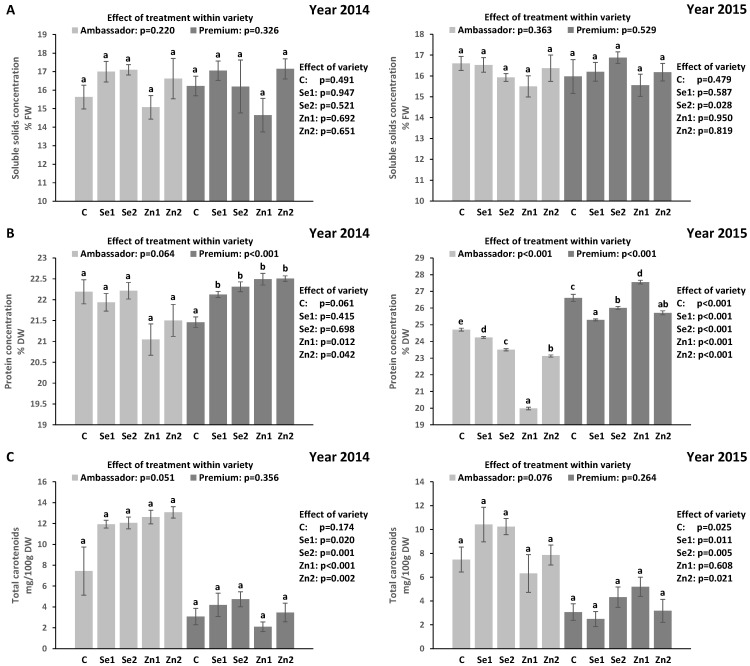
Effect of foliar Se and Zn treatments and variety in two growing seasons (2014 and 2015) on the concentration of soluble solids (**A**), protein (**B**) and total carotenoid (**C**) in pea seeds. C (control): without Se and Zn; Se1: 50 g Se/ha; Se2: 100 g Se/ha; Zn1: 375 g Zn/ha; Zn2: 750 g Zn/ha; mean ± SD; *n* = 4. Bars not sharing the same lowercase letters (superscripts) are significantly different within variety. *p*-values on the right side of the figure show the effect of treatment across the two varieties (i.e., Ambassador vs. Premium).

For both years, treatment did not significantly affect SSC vs. controls in Ambassador and Premium. Premium showed significantly higher SSC than Ambassador only for the Se2 treatment in the 2015 growing season (Figure 1A). Growing year had no significant effect on SSC in both varieties (*p* > 0.05).

### 3.4. Protein Concentration in Seeds

In 2014, treatment did not significantly affect protein concentration vs. the control in Ambassador, while all Se and Zn treatments significantly increased protein concentration vs. the control in Premium. Premium showed significantly higher protein concentration than Ambassador only for Zn1 and Zn2. In 2015, all Se and Zn treatments significantly decreased protein concentration vs. the control in Ambassador, while the Zn1 treatment significantly increased, and Se1, Se2 and Zn2 significantly decreased protein concentration vs. the control in Premium. Premium showed significantly higher protein concentration than Ambassador for all treatments (Figure 1B). Growing year had a significant effect on protein concentration in both varieties (*p* < 0.001).

### 3.5. Total Carotenoid Concentration in Seeds

In 2014, treatment failed to show a significant effect (*p* = 0.051) on total carotenoid concentration vs. the control in Ambassador, while it did not significantly influence total carotenoid concentration vs. the control in Premium. Ambassador showed significantly higher total carotenoid concentration than Premium for all treatments except for the control. In 2015, treatment did not significantly affect total carotenoid concentration vs. controls in both varieties. Ambassador showed significantly higher total carotenoid concentration than Premium for all treatments except for Zn1 (Figure 1C). Growing year had a significant effect on total carotenoid concentration in Ambassador (*p* = 0.010) compared with Premium (*p* = 0.823).

### 3.6. Chlorophyll a, Chlorophyll b and Total Chlorophyll Concentration in Seeds

In 2014, treatment did not significantly affect chlorophyll a concentration vs. controls in both varieties. Ambassador showed significantly higher chlorophyll a concentration than Premium only for Zn2, while Premium showed a trend for Se1 treatment. In 2015, treatment did not significantly influence chlorophyll a concentration vs. the control in Ambassador, while the Zn1 treatment significantly increased chlorophyll a concentration vs. the control in Premium. Ambassador showed significantly higher chlorophyll a concentration than Premium for all treatments except for Zn1. Growing year had a significant effect on chlorophyll a concentration in both varieties (Table 3).

For both years, treatment did not significantly affect chlorophyll b concentration vs. controls in Ambassador and Premium. Premium showed significantly higher chlorophyll b concentration than Ambassador only for the Se1 treatment in the 2014 growing season. Growing year had no significant effect on chlorophyll b concentration in both varieties (Table 3).

For both years, treatment did not significantly influence total chlorophyll concentration vs. controls in Ambassador and Premium. Premium showed significantly higher total chlorophyll concentration than Ambassador only for the Se1 treatment in the 2014 growing season. In contrast, Ambassador showed significantly higher total chlorophyll concentration than Premium only for Zn2 in 2014 and for the control in the 2015 growing season. Growing year had a significant effect on total chlorophyll concentration in Premium compared with Ambassador (Table 3).

### 3.7. Total Condensed Tannin Concentration in Seeds

For both years, treatment did not significantly affect total condensed tannins vs. controls in Ambassador and Premium, and no significant differences were found for total condensed tannins between Ambassador and Premium for all treatments. Growing year had no significant effect on total condensed tannins in both varieties (Table 3).

### 3.8. Correlations

For Ambassador seeds, chlorophyll a concentration was positively correlated with chlorophyll b concentration and total chlorophyll concentration. Chlorophyll b concentration showed a positive correlation with total chlorophyll concentration. Zn concentration was positively correlated with Mg concentration and protein concentration. For Premium seeds, Ca concentration was negatively correlated with Mg concentration, K concentration and protein concentration. Mg concentration showed a positive correlation with K concentration and protein concentration, and a negative correlation with chlorophyll a concentration. A positive correlation was observed between K concentration and Na concentration. Chlorophyll a concentration was positively correlated with total chlorophyll concentration and negatively correlated with protein concentration. A positive correlation was found between chlorophyll b concentration and total chlorophyll concentration. Zn concentration was positively correlated with Mg concentration and protein concentration and negatively correlated with Ca concentration and chlorophyll a concentration (Table 4).

For Ambassador seeds, total condensed tannins were significantly and positively correlated with SSC (r^2^ = 0.131), chlorophyll b concentration (r^2^ = 0.128) and total chlorophyll concentration (r^2^ = 0.109). For Premium seeds, total condensed tannins were not significantly correlated with other parameters (Table 5).

For Ambassador seeds, FRAP was positively correlated with K concentration (r^2^ = 0.109) and protein concentration (r^2^ = 0.123), while it was negatively correlated with Ca concentration (r^2^ = 0.239). For Premium seeds, ABTS was positively correlated with protein concentration (r^2^ = 0.355) and total condensed tannins (r^2^ = 0.157), while it was negatively correlated with chlorophyll a concentration (r^2^ = 0.317) and total chlorophyll concentration (r^2^ = 0.126). FRAP showed a positive correlation with chlorophyll a concentration (r^2^ = 0.276), and a negative correlation with Mg (r^2^ = 0.202) and protein concentrations (r^2^ = 0.367) (Table S2).

For Ambassador, Mg concentration was significantly and negatively correlated with seed dry matter (r^2^ = 0.360), number of seeds/pod (r^2^ = 0.241), pod length (r^2^ = 0.230) and pod perimeter (r^2^ = 0.516). A significant and negative correlation was also found between protein concentration and number of seeds/pod (r^2^ = 0.124) and pod perimeter (r^2^ = 0.249). Na concentration showed a significant and positive correlation with seed dry matter (r^2^ = 0.245). Chlorophyll a concentration was significantly and positively correlated with seed dry matter (r^2^ = 0.116), number of seeds/pod (r^2^ = 0,176) and pod perimeter (r^2^ = 0.119). A significant and positive correlation was also found between total condensed tannins and pod length (r^2^ = 0.106) and pod perimeter (r^2^ = 0.199). For Premium, Ca concentration was significantly and positively correlated with seed dry matter (r^2^ = 0.170) and pod perimeter (r^2^ = 0.282). Chlorophyll a concentration showed a significant and positive correlation with seed dry matter (r^2^ = 0.191) and pod perimeter (r^2^ = 0.605). A significant and positive correlation was also found between total chlorophyll concentration and pod perimeter (r^2^ = 0.205). Mg concentration was significantly and negatively correlated with seed dry matter (r^2^ = 0.187) and pod perimeter (r^2^ = 0.484). A significant and negative correlation was also found between protein concentration and seed dry matter (r^2^ = 0.315) and pod perimeter (r^2^ = 0.733) (Table S3).

## 4. Discussion

In the present work, the impact of foliar-applied selenate and zinc oxide at the flowering stage on two pea varieties (Ambassador and Premium) was studied, and the concentrations of selected macrominerals, macronutrients and bioactive compounds were evaluated in seeds. Treatments had no beneficial effect on concentrations of macrominerals, while they, in part, though not consistently, enhanced concentrations of Mg, protein and chlorophyll a (Table 1, Table 2 and Table 3 and Figure 1).

Pea was used in the study, as it is an important legume and staple food crop, contributing to food security. Ambassador and Premium were employed due to their high-yielding performance. In addition, previous studies demonstrated that dark-seeded varieties of pea have a large capacity for accumulating phenolics [26,27,28,29]. Selenium and zinc solutions were foliar applied as this strategy limited the impact of soil conditions on interactions between the examined elements. Foliar fertilization with selenate was employed due to its high effectiveness [30]. While not essential for higher plants, Se at low concentrations may positively affect plant growth, development and yield, and improves resistance to abiotic stresses [31]. Zinc oxide was examined in accordance with agro-industry recommendations. In contrast to Se, Zn is an essential trace element for plant life [32]. Macrominerals, macronutrients and bioactive compounds were investigated, which are important for plant growth and/or are associated with human health effects.

Our research already revealed that foliar application of sodium selenate enhanced seed Se accumulation in both pea varieties dose-dependently. Premium variety showed greater ability to accumulate Se in seeds than Ambassador variety. Highest Se accumulation was reported in seeds of Premium upon application of 100 g Se/ha (7.84 mg/kg vs. the control (0.16 mg/kg), DW). Contrarily, seed Zn accumulation was not significantly influenced by the foliar application of zinc oxide (unpublished results) (Table S1).

In the present study, selenate and zinc oxide treatments had no significant effects on concentrations of Ca, Mg, K and Na in pea seeds (Table 1 and Table 2). In contrast, Poblaciones and Rengel [33] showed that foliar-applied selenate and zinc sulfate individually and at various combinations at early seed filling of field pea did not significantly influence seed concentrations of Ca and Mg. Another study by Poblaciones and Rengel [34] reported that foliar selenate treatments at the start of seed filling of field pea significantly decreased seed concentrations of Ca and Mg (vs. the control). Poblaciones and Rengel [35], whose work aimed to investigate the influence of all combinations of soil application and foliar application (before flowering and at early seed-filling stage) of zinc sulfate on field pea, found that seed Mg concentration was significantly reduced only by the soil Zn application (vs. the control), while seed Ca concentration was not affected by any of the examined conditions.

The present study further showed synergistic relationships between Mg and Zn concentration (for Ambassador and Premium seed), Mg and K concentration (for Premium seed) and between K and Na concentration (for Premium seed). Inverse relationships were found between Ca and Mg, as well as between K and Zn concentrations (for Premium seed) (Table 4). The relationship between minerals in the plant may be influenced by numerous factors, including plant species and genotype, dose and form of fertilizer, method of fertilizer application, cultivation conditions, soil characteristics [36,37,38,39], and antagonism and/or synergism between various elements [40,41].

Selenate treatments had no beneficial effect on the concentration of soluble solids in pea seed (Figure 1A). To the best of our knowledge, there is a gap regarding research on carbohydrate metabolism in legumes (including pea) upon foliar Se application. Selenium accumulation may disturb or promote carbohydrate metabolism, depending on the concentration and form of applied Se, as well as the development stage of the plant as reviewed by Lara et al. [42]. Our study further showed that zinc oxide treatments did not promote the accumulation of soluble solids in pea seed (Figure 1A). In contrast, previous studies reported a beneficial influence of foliar-applied zinc sulfate on the concentration of total sugars in seeds of pea [43] and black gram [44]. Another study found both non-significant and beneficial effects of foliar-applied zinc sulfate on the concentration of total sugars in seeds of common bean [45]. In the present study, non-beneficial effects of Se treatments on soluble solids concentration in pea seed (Figure 1A) may be associated with reduced seed chlorophyll concentration (Table 3). Carbohydrates act as an energy source needed for the development of pollen. Low Zn concentration has been associated with poor photosynthesis, which decreases synthesis of sugars required for growth and seed development. Reduced activity of sucrose synthase, an enzyme involved in sugar synthesis that plays an essential role in seed filling and seed size, has also been found under Zn deficiency conditions, as reviewed by Pandey et al. [43].

Selenate and zinc oxide partly enhanced protein concentration in Premium seeds. The highest protein concentration was found in seeds of Premium treated with 375 g Zn/ha (27.6% DW) vs. the control (26.6% DW) (Figure 1B). In contrast, Poblaciones et al. [46] showed that protein concentration in pea seed was not affected by foliar-applied Se doses (at the flowering stage), while it was affected by the Se form. Authors found that selenite was rather less effective for improving seed protein concentration than selenate, and indicated that this may result from the fact that selenite exerts generally more toxic effects on plants than selenate. Similarly, Poblaciones and Rengel [34] did not find a significant effect of foliar-applied selenate (at the start of seed filling) on protein concentration in pea seed. Individual and combined foliar application of selenate and zinc sulfate at early seed filling of pea also had no significant influence on seed protein concentration [33]. However, another study by Poblaciones and Rengel [35] showed a beneficial effect of foliar-applied zinc sulfate (before flowering and at early seed-filling stage) on protein concentration in pea seed compared to soil and combined (soil + foliar) application of zinc sulfate.

Furthermore, the present study did not find a significant correlation between seed Se concentration and seed protein concentration for both varieties (*p* > 0.05, data not shown). In contrast, seed Zn concentration showed a significant and positive correlation with seed protein concentration for both varieties, with a stronger correlation reported for Premium compared to Ambassador (Table 4). Previous research on foliar Se and Zn fertilization indicated that field peas, mainly due to their higher protein concentration, may possess a greater potential for Se and Zn uptake and seed accumulation than cereals [33,34]. Zinc is essential for protein synthesis due to its role in DNA/RNA metabolism, chromatin disposition and gene expression, as reviewed by Moreira et al. [47]. Regarding Se, it has been revealed that the predominant Se species identified in pea seeds upon foliar application of selenate was selenomethionine, an organic Se compound beneficial for human and animal health, recognized for its strong antioxidant activity [48].

Selenate and zinc oxide did not significantly affect chlorophyll a and b, total chlorophyll and total carotenoid concentrations except for 375 g Zn/ha (21.5 mg/100g DW) that significantly increased chlorophyll a concentration vs. the control (10.7 mg/100g DW) in Premium seeds (from 2015 growing season) (Table 3, Figure 1C). We can merely hypothesize on the relation between Zn and chlorophyll/carotenoid accumulation. Carbonic anhydrases (CAs) are zinc metalloenzymes that catalyze the interconversion of CO_2_ and HCO_3_¯ and are essential for photosynthetic organisms; moreover, CAs are clearly important in the photosynthesis process [49]. For example, rice leaves treated with foliar Zn showed increased activity of CA, and hence increased photosynthesis [50]. Inhibition of photosynthesis induced by Zn deficiency may be associated with a decrease in CA activity, the photochemical activity of chloroplasts, chlorophyll concentration, as well as alterations in chloroplast structure [51]. Furthermore, carotenoids are typically associated with chlorophylls within the light harvesting complex I/II, thus zinc may upregulate photosynthetic processes and pigment density [52,53]. It has been found that selenium treatment (as sodium selenate), under hydroponic culture conditions, decreased carotenoid and chlorophyll accumulation in arabidopsis (*Arabidopsis thaliana*) compared to controls. The authors indicated that a selenium-induced decrease in carotenoid biosynthesis took place through the downregulation of phytoene synthase at the beginning of the carotenoid biosynthesis pathway. Differential expression of divinyl chlorophyll vinyl reductase (DVR) (upregulation of the AT4G35250 gene) involved in the chlorophyll a biosynthesis pathway was also shown in Se-treated arabidopsis plants [54]. It indicates that foliar Se treatment may differently affect other genes and enzymes responsible for carotenoid and chlorophyll biosynthesis in pea seeds.

Concentration of total condensed tannins in pea seeds was not significantly affected by selenate and zinc oxide treatments (Table 1 and Table 3). To the best of our knowledge, the effect of foliar Se/Zn application on tannin accumulation in legumes, including pea, has not been investigated so far. Song et al. [55] found that foliar zinc sulfate application enhanced the accumulation of phenolic compounds including tannins, as well as total soluble solids in grape berries growing on Zn deficient soil. Another study found that key genes for polyphenol formation, namely DFR and LDOX, catalyzed the formation of certain phenolics, such as proanthocyanidins and anthocyanidins [56,57]. According to Song et al. [55], foliar zinc sulfate application could significantly influence the expression of phenolic (especially flavonol and anthocyanin) biosynthesis pathway genes (VvPAL, VvSTS29, VvCHS, VvCHI, VvF3H, VvFLS4, VvDFR, VvLDOX and VvMYBF1) throughout berry development, consequently supporting phenolic accumulation. The study further indicated that sucrose is a positive regulator of the biosynthesis of phenolics (especially flavonoids) [58], and the beneficial impact of Zn treatment on photosynthesis and sugar accumulation may possibly enhance flavonoid biosynthesis.

Tannins are present in many bushes and trees, and also in the majority of legume seeds, especially in coloured seed coats. These compounds can diminish the nutritional value of legumes by reducing the digestibility and bioavailability of nutrients, including minerals. Tannins can interact especially with divalent minerals such as iron, calcium, magnesium and zinc, and to a lesser extent interacts with potassium and sodium, mainly by chelation and inhibition of digestive enzymes, which eventually lead to reduced availability of minerals for absorption in the gastrointestinal tract, as reviewed by Grela et al. [59]. In the present study, total condensed tannins did not compromise accumulation of minerals, macronutrients and bioactive compounds in pea seeds (Table 5), which is promising regarding agronomic biofortification. It is also worth stressing that food processing methods, such as soaking and cooking, may improve the nutritional value of legumes including peas by decreasing or removing anti-nutrients such as tannins, phytic acid and trypsin inhibitors, as reviewed previously [35,60].

Finally, to the best of our knowledge, correlations between minerals, macronutrients, bioactive plant compounds and total antioxidant activity of pea have scantly been investigated so far. The lack of significant and strong correlations (Table S2) indicated that other compounds may have contributed more substantially to antioxidant mechanisms in pea [61].

## 5. Conclusions

Our study has shown that foliar application of sodium selenate and zinc oxide at the flowering stage enhanced, in part, Mg concentrations in Ambassador seed and chlorophyll a concentrations in Premium seed, respectively. Selenate and zinc oxide applications also improved, in part, protein concentrations in Premium seed. Highest protein concentration was found in seeds of Premium variety treated with 375 g Zn/ha, though seed Zn concentration was not influenced by zinc oxide treatments. Total condensed tannins did not compromise accumulation of all determined minerals, macronutrients and bioactive compounds in seeds of both pea varieties. The evaluation of the impact of climate conditions on the accumulation of macrominerals, macronutrients and bioactive compounds in pea seeds upon foliar application of Se/Zn requires further experiments under field conditions. Further research in this domain should include studying broader concentration ranges of foliar selenate and zinc oxide, in addition to other forms of zinc, such as zinc sulfate in combination with additional pea varieties.

## Figures and Tables

**Table 1:** Combined analysis of variance for the effects of year, variety and treatment on seed Ca, Mg, K, Na, soluble solid, protein, chlorophyll a, chlorophyll b, total chlorophyll, total condensed tannin and total carotenoid concentrations.

	Year (Y)	Variety (V)	Treatment (T)	Y × V	Y × T	V × T	Y × V × T
DF	1	1	4	1	4	4	4
Ca (mg/kg DW)	<0.001	<0.001	NS	0.053	NS	NS	NS
Mg (mg/kg DW)	<0.001	<0.001	NS	0.020	NS	NS	NS
K (mg/kg DW)	0.027	<0.001	NS	NS	NS	NS	NS
Na (mg/kg DW)	<0.001	0.051	NS	<0.001	NS	NS	NS
Soluble solids (% FW)	NS	NS	0.014	NS	NS	NS	NS
Protein (% DW)	<0.001	<0.001	<0.001	<0.001	<0.001	<0.001	<0.001
Chlorophyll a (mg/100 g DW)	<0.001	<0.001	NS	<0.001	NS	0.023	0.003
Chlorophyll b (mg/100 g DW)	NS	NS	NS	NS	NS	NS	NS
Total chlorophyll (mg/100 g DW)	0.005	0.006	NS	NS	NS	0.054	NS
Total carotenoids (mg/100 g DW)	NS	<0.001	NS	NS	NS	NS	0.025
TCT (mg/100 g DW)	NS	NS	NS	NS	NS	NS	NS

TCT: total condensed tannins; DF: degrees of freedom; NS: not significant.

**Table 2:** Effect of foliar-applied Se and Zn, variety and year on seed Ca, Mg, K and Na concentrations.

		Ca (mg/kg DW)			Mg (mg/kg DW)			K (mg/kg DW)			Na (mg/kg DW)		
Year	Treatment	Ambassador	Premium	*p*-Value	Ambassador	Premium	*p*-Value	Ambassador	Premium	*p*-Value	Ambassador	Premium	*p*-Value
2014	Control	903 ± 45.8	1574 ± 320	**0.004**	1247 ± 39.4	1152 ± 37.3	**0.012**	13,539 ± 426	11,254 ± 1128	**0.010**	92.2 ± 86.5	45.2 ± 11.3	0.283
	Se1	953 ± 80.9	1515 ± 155	**<0.001**	1249 ± 72.4	1135 ± 72.6	0.068	13,530 ± 489	11,695 ± 1222	**0.033**	48.7 ± 10.1	54.4 ± 13.2	0.571
	Se2	954 ± 40.3	1577 ± 205	**<0.001**	1247 ± 52.7	1149 ± 108	0.142	13,839 ± 973	11,505 ± 1236	**0.023**	59.3 ± 26.0	44.8 ± 14.8	0.359
	Zn1	896 ± 89.4	1545 ± 187	**<0.001**	1257 ± 14.3	1151 ± 152	0.196	14,175 ± 597	11,865 ± 1259	**0.017**	41.2 ± 6.69	47.2 ± 13.2	0.494
	Zn2	965 ± 97.9	1484 ± 146	**0.001**	1234 ± 20.0	1143 ± 97.1	0.109	13,208 ± 973	11,314 ± 880	**0.028**	55.2 ± 8.98	49.8 ± 4.43	0.359
	*p*-value	0.582	0.983		0.970	0.999		0.448	0.932		0.446	0.756	
2015	Control	910 ± 52.2 ^AB^	1308 ± 13.3	**<0.001**	1382 ± 32.8 ^A^	1441 ± 25.5 ^C^	**0.029**	13,999 ± 612	12,393 ± 485 ^B^	**0.006**	31.2 ± 3.43	48.8 ± 7.27	**0.003**
	Se1	808 ± 76.2 ^A^	1379 ± 56.5	**<0.001**	1455 ± 7.02 ^B^	1341 ± 10.0 ^AB^	**<0.001**	14,426 ± 529	11,802 ± 223 ^AB^	**<0.001**	36.1 ± 8.67	50.8 ± 11.7	0.084
	Se2	844 ± 36.1 ^A^	1316 ± 41.1	**<0.001**	1418 ± 24.2 ^AB^	1380 ± 29.2 ^B^	0.095	14,405 ± 609	11,751 ± 217 ^AB^	**<0.001**	31.9 ± 3.48	43.2 ± 11.0	0.088
	Zn1	988 ± 89.9 ^B^	1334 ± 57.6	**0.001**	1411 ± 45.8 ^AB^	1333 ± 19.6 ^A^	**0.017**	13,424 ± 1140	12,187 ± 174 ^AB^	0.068	37.4 ± 14.4	48.5 ± 3.26	0.130
	Zn2	917 ± 22.0 ^AB^	1392 ± 30.6	**<0.001**	1386 ± 11.2 ^A^	1314 ± 16.7 ^A^	**<0.001**	13,745 ± 533	11,578 ± 412 ^A^	**0.001**	28.8 ± 4.26	46.0 ± 4.23	**0.001**
	*p*-value	**0.006**	0.050		**0.015**	**<0.001**		0.259	**0.016**		0.566	0.683	
	* *p*-value across	0.059	**0.001**		**<0.001**	**<0.001**		0.153	0.094		**<0.001**	0.966	

Control: without Se/Zn; Se1: 50 g Se/ha; Se2: 100 g Se/ha; Zn1: 375 g Zn/ha; Zn2: 750 g Zn/ha; mean ± SD; *n* = 4. Means within a column followed by different letters are significantly different. *p*-values in the same row mean the effect of Se/Zn dose. *p*-values in the same column mean the effect of variety. * *p*-values across refer to the effect of year. *p*-values in bold are statistically significant.

**Table 3:** Effect of foliar-applied Se and Zn, variety and year on seed chlorophyll a, chlorophyll b, total chlorophyll and total condensed tannin concentrations.

		Chlorophyll a (mg/100 g DW)			Chlorophyll b (mg/100 g DW)			TCH (mg/100 g DW)			TCT (mg/100 g DW)		
Year	Treatment	Ambassador	Premium	*p*-Value	Ambassador	Premium	*p*-Value	Ambassador	Premium	*p*-Value	Ambassador	Premium	*p*-Value
2014	Control	32.7 ± 6.28	26.4 ± 4.31	0.149	20.8 ± 13.6	7.55 ± 7.20	0.137	53.5 ± 19.8	33.9 ± 11.4	0.138	667 ± 84.5	1174 ± 59.8	0.843
	Se1	29.2 ± 2.15	34.5 ± 3.81	0.051	10.6 ± 0.81	17.7 ± 5.60	**0.045**	39.7 ± 2.92	52.2 ± 8.99	**0.039**	652 ± 99.6	1233 ± 63.0	0.385
	Se2	30.4 ± 3.72	30.6 ± 5.90	0.974	11.0 ± 1.53	11.7 ± 7.44	0.866	41.5 ± 5.19	42.3 ± 13.3	0.915	760 ± 109	1160 ± 73.4	0.473
	Zn1	31.3 ± 2.22	26.6 ± 4.13	0.095	11.5 ± 0.80	10.5 ± 6.44	0.770	42.7 ± 3.01	37.1 ± 10.4	0.338	787 ± 187	1213 ± 81.4	0.994
	Zn2	33.0 ± 2.24	27.9 ± 1.65	**0.010**	12.4 ± 0.79	10.2 ± 3.44	0.255	45.5 ± 2.97	38.1 ± 4.61	**0.037**	788 ± 91.9	1333 ± 173	0.449
	*p*-value	0.574	0.076		0.160	0.256		0.313	0.156		0.797	0.495	
2015	Control	30.2 ± 1.76	10.7 ± 4.04 ^A^	**<0.001**	14.7 ± 5.73	8.52 ± 6.84	0.213	44.9 ± 7.34	19.3 ± 10.9	**0.008**	956 ± 212	793 ± 151	0.468
	Se1	27.2 ± 6.60	12.5 ± 4.37 ^AB^	**0.010**	13.2 ± 11.9	11.5 ± 7.38	0.814	40.5 ± 18.4	24.0 ± 11.7	0.182	945 ± 272	862 ± 73.9	0.902
	Se2	27.7 ± 9.17	14.2 ± 3.61 ^AB^	**0.034**	16.1 ± 16.1	11.9 ± 5.96	0.642	43.8 ± 25.2	26.1 ± 9.57	0.238	993 ± 39.3	823 ± 210	0.747
	Zn1	21.3 ± 3.80	21.5 ± 6.13 ^B^	0.957	12.4 ± 7.13	22.8 ± 11.5	0.173	33.7 ± 10.9	44.3 ± 17.6	0.343	923 ± 70.1	933 ± 39.7	0.250
	Zn2	27.3 ± 4.34	16.9 ± 2.33 ^AB^	**0.006**	12.7 ± 5.12	14.8 ± 4.84	0.560	40.0 ± 9.32	31.8 ± 7.13	0.213	1047 ± 266	891 ± 66.2	0.419
	*p*-value	0.308	**0.023**		0.983	0.141		0.864	0.077		0.949	0.719	
	* *p*-value across	**0.005**	**<0.001**		0.832	0.286		0.334	**0.002**		0.365	0.291	

Control: without Se/Zn; Se1: 50 g Se/ha; Se2: 100 g Se/ha; Zn1: 375 g Zn/ha; Zn2: 750 g Zn/ha; mean ± SD; *n* = 4; TCH: total chlorophyll concentration; TCT: total condensed tannins. Means within a column followed by different letters are significantly different. *p*-values in the same row mean the effect of Se/Zn dose. *p*-values in the same column mean the effect of variety. * *p*-values across refer to the effect of year. p-values on bold represent statistical significant findings.

**Table 4:** Results of regression analysis between minerals, protein and chlorophylls investigated for seed of two pea varieties (Ambassador and Premium).

Variety	Correlations	Regression Equation	r^2^
Ambassador			
	Chla vs. Chlb	y = 1.0861x − 18.013	0.544
	Chla vs. TCH	y = 2.0861x − 18.013	0.815
	Chlb vs. TCH	y = 1.5011x + 22.261	0.915
	Mg vs. Zn	y = 0.0834x − 65.672	0.735
	Zn vs. protein	y = 4.036x − 45.495	0.437
Premium			
	Ca vs. Mg	y = −0.5834x + 2095.3	0.573
	Ca vs. K	y = −3.5624x + 16873	0.545
	Ca vs. protein	y = −0.0078x + 35.448	0.375
	Mg vs. K	y = 3.9935x + 6727.3	0.407
	Mg vs. Chla	y = −0.0478x + 82.169	0.513
	Mg vs. protein	y = 0.0136x + 7.1227	0.682
	K vs. Na	y = 0.0068x − 32.48	0.344
	Chla vs. TCH	y = 1.3569x + 4.8012	0.749
	Chlb vs. TCH	y = 1.479x + 16.094	0.663
	Chla vs. protein	y = −0.1763x + 28.118	0.509
	Zn vs. Ca	y = −0.0357x + 87.093	0.483
	Zn vs. Mg	y = 0.0599x − 39.455	0.807
	Zn vs. Chla	y = −0.7707x + 52.691	0.597
	Zn vs. protein	y = 3.7961x − 56.303	0.884

Chla: chlorophyll a concentration; Chlb: chlorophyll b concentration; TCH: total chlorophyll concentration. Results significant at *p* < 0.001.

**Table 5:** Pearson correlation coefficients between the concentrations of total condensed tannins and minerals, macronutrients and bioactive compounds evaluated for seeds of two pea varieties (Ambassador and Premium).

Constituent	Ambassador	Premium
Se	−0.007	−0.036
Zn	0.167	0.004
Ca	0.172	0.056
Mg	0.163	−0.174
K	0.284	−0.203
Na	−0.037	−0.006
SSC	0.362 *	−0.138
Protein	−0.015	0.041
Chla	0.236	−0.008
Chlb	0.358 *	0.113
TCH	0.330 *	0.057
TCC	−0.228	0.210

SSC: soluble solids concentration; Chla: chlorophyll a concentration; Chlb: chlorophyll b concentration; TCH: total chlorophyll concentration; TCC: total carotenoid concentration. Level of significance: * *p* < 0.05.

## Data Availability

Not applicable.

## Supplementary Materials

The following supporting information can be downloaded at: https://www.mdpi.com/article/10.3390/plants11152009/s1, Table S1: Effect of foliar-applied Se and Zn, variety and year on the seed Se and Zn concentrations; Table S2: Pearson correlation coefficients between total antioxidant activity (ABTS and FRAP) and macrominerals, macronutrients and bioactive compounds evaluated for seeds of two pea varieties (Ambassador and Premium); Table S3: Pearson correlation coefficients between growth parameters and macrominerals, macronutrients and bioactive compounds evaluated for seeds of two pea varieties (Ambassador and Premium).
