# Supplementary material for: Separate Effects of Foliar Applied Selenate and Zinc Oxide on the Accumulation of Macrominerals, Macronutrients and Bioactive Compounds in Two Pea (Pisum sativum L.) Seed Varieties
Source: Plants (Basel). 2022 Aug 1;11(15):2009. doi: 10.3390/plants11152009 (PMC9370774; doi:10.3390/plants11152009)
Supplement: Supplementary file 1 [file plants-11-02009-s001.zip › plants-1799121-supplementary.pdf]

plants

MDPI

Supplementary Material

Table S1. Effect of foliar-applied Se and Zn, variety and year on seed Se and Zn concentrations.

		Se (mg/kg DW)			Zn (mg/kg DW)		
**Year**	**Treatment**	**Ambassador**	**Premium**	***p*-Value**	**Ambassador**	**Premium**	***p*-Value**
2014	Control	0.08 ± 0.01 ^{A}	0.16 ± 0.06 ^{A}	**0.039**	41.6 ± 3.47	25.8 ± 3.15	**0.001**
	Se1	2.59 ± 0.45 ^{B}	4.28 ± 1.69 ^{B}	0.180	34.4 ± 1.64	25.2 ± 2.87	**0.002**
	Se2	4.87 ± 3.20 ^{B}	7.84 ± 2.97 ^{B}	0.323	38.2 ± 6.15	26.9 ± 1.77	**0.008**
	Zn1	0.09 ± 0.01 ^{A}	0.18 ± 0.06 ^{A}	**0.013**	38.5 ± 2.45	30.1 ± 5.36	**0.027**
	Zn2	0.08 ± 0.01 ^{A}	0.18 ± 0.07 ^{A}	**0.031**	35.4 ± 1.25	29.4 ± 3.46	**0.018**
	p-value	**< 0.001**	**< 0.001**		0.076	0.216	
2015	Control	0.12 ± 0.02 ^{A}	0.09 ± 0.02 ^{A}	**0.033**	55.3 ± 1.97 ^{A}	44.9 ± 2.18 ^{BC}	**< 0.001**
	Se1	1.26 ± 0.04 ^{CD}	3.14 ± 0.11 ^{D}	**< 0.001**	55.7 ± 1.62 ^{A}	41.3 ± 0.42 ^{A}	**< 0.001**
	Se2	2.99 ± 0.08 ^{D}	5.76 ± 0.10 ^{E}	**< 0.001**	54.9 ± 1.52 ^{A}	42.8 ± 0.74 ^{AB}	**< 0.001**
	Zn1	0.90 ± 1.33 ^{BC}	0.23 ± 0.01 ^{C}	0.338	45.0 ± 7.49 ^{B}	44.2 ± 0.28 ^{BC}	0.918
	Zn2	0.15 ± 0.01 ^{AB}	0.13 ± 0.02 ^{B}	**< 0.001**	52.0 ± 1.15 ^{AB}	45.3 ± 1.10 ^{C}	0.051
	p-value	**< 0.001**	**< 0.001**		**0.003**	**< 0.001**	
	* *p*-value across	0.074	0.097		**< 0.001**	**< 0.001**	

Control: without Se/Zn; Se1: 50 g Se/ha; Se2: 100 g Se/ha; Zn1: 375 g Zn/ha; Zn2: 750 g Zn/ha; mean ± SD; $n = 4$. Means within a column followed by different letters are significantly different. *p*-values in the same row mean the effect of Se/Zn dose. *p*-values in the same column mean the effect of variety. * *p*-values across refer to the effect of year.

Table S2. Pearson correlation coefficients between total antioxidant activity (ABTS and FRAP) and macrominerals, macronutrients and bioactive compounds evaluated for seeds of two pea varieties (Ambassador and Premium).

**Variety/ Antioxidant Test**	**Ca**	**Mg**	**K**	**Na**	**SSC**	**Protein**	**Chla**	**Chlb**	**TCH**	**TCC**	**TCT**
Ambassador											
ABTS	0.081	0.281	0.156	0.264	0.250	0.211	0.034	0.073	0.062	−0.110	0.097
FRAP	−0.489 **	0.169	0.331 *	0.093	0.080	0.350 *	−0.017	−0.024	−0.023	0.115	−0.076
Premium											
ABTS	−0.291	0.293	−0.173	−0.227	−0.213	0.596 ***	−0.563 ***	0.008	−0.355 *	0.240	0.397 *
FRAP	0.195	−0.450 **	0.166	0.291	0.245	−0.606 ***	0.525 ***	−0.177	0.237	−0.062	−0.073

SSC: soluble solids concentration; Chla: chlorophyll a concentration; Chlb: chlorophyll b concentration; TCH: total chlorophyll concentration; TCC: total carotenoid concentration; TCT: total condensed tannin concentration. Level of significance: * $p < 0.05$, ** $p < 0.01$, *** $p < 0.001$.

Table S3. Pearson correlation coefficients between growth parameters and macrominerals, macronutrients and bioactive compounds evaluated for seeds of two pea varieties (Ambassador and Premium).

**Variety/ Growth Parameter**	**Ca**	**Mg**	**K**	**Na**	**SSC**	**Protein**	**Chla**	**Chlb**	**TCH**	**TCC**	**TCT**
Ambassador											
Seed dry matter	0.290	−0.600 **	−0.210	0.495 **	0.189	−0.093	0.340 *	0.042	0.174	0.266	−0.058
Number of seeds/pod	0.080	−0.491 **	0.005	0.096	−0.178	−0.352 *	0.419 **	0.065	0.223	0.280	0.025
Pod length	0.165	−0.480 **	−0.119	0.245	0.176	−0.174	0.126	−0.168	−0.052	0.326 *	−0.037
Pod perimeter	0.311	−0.718 **	−0.095	0.308	0.045	−0.499 **	0.345 *	−0.009	0.144	0.446 **	−0.048
Premium											
Seed dry matter	0.412 **	−0.433 **	−0.095	−0.086	−0.045	−0.561 **	0.437 **	−0.121	0.212	−0.046	−0.119
Number of seeds/pod	0.191	−0.079	−0.039	0.093	0.240	−0.295	0.195	−0.094	0.073	−0.032	−0.185
Pod length	−0.045	0.265	0.042	−0.090	0.106	0.154	−0.257	−0.048	−0.191	−0.041	−0.160
Pod perimeter	0.531 **	−0.696 **	−0.179	0.040	0.067	−0.856 **	0.778 **	−0.078	0.453 **	0.072	−0.081

SSC: soluble solids concentration; Chla: chlorophyll a concentration; Chlb: chlorophyll b concentration; TCH: total chlorophyll concentration; TCC: total carotenoid concentration; TCT: total condensed tannin concentration. Level of significance: * $p < 0.05$, ** $p < 0.01$.
